# Application of an alchemical free energy method for the prediction of thermostable DuraPETase variants

**DOI:** 10.1007/s00253-024-13144-z

**Published:** 2024-04-21

**Authors:** Sebastian Schreiber, David Gercke, Florian Lenz, Joachim Jose

**Affiliations:** University of Münster, Institute of Pharmaceutical and Medicinal Chemistry, PharmaCampus, Corrensstr. 48, 48149 Münster, Germany

**Keywords:** Alchemical free energy calculations, Enzyme engineering, Thermostability, MD simulation, PETase

## Abstract

**Abstract:**

Non-equilibrium (NEQ) alchemical free energy calculations are an emerging tool for accurately predicting changes in protein folding free energy resulting from amino acid mutations. In this study, this method in combination with the Rosetta *ddg monomer* tool was applied to predict more thermostable variants of the polyethylene terephthalate (PET) degrading enzyme DuraPETase. The Rosetta *ddg monomer* tool efficiently enriched promising mutations prior to more accurate prediction by NEQ alchemical free energy calculations. The relative change in folding free energy of 96 single amino acid mutations was calculated by NEQ alchemical free energy calculation. Experimental validation of ten of the highest scoring variants identified two mutations (DuraPETase^S61M^ and DuraPETase^S223Y^) that increased the melting temperature (*T*_m_) of the enzyme by up to 1 °C. The calculated relative change in folding free energy showed an excellent correlation with experimentally determined *T*_m_ resulting in a Pearson’s correlation coefficient of *r* =  − 0.84. Limitations in the prediction of strongly stabilizing mutations were, however, encountered and are discussed. Despite these challenges, this study demonstrates the practical applicability of NEQ alchemical free energy calculations in prospective enzyme engineering projects.

**Key points:**

*• Rosetta ddg monomer enriches stabilizing mutations in a library of DuraPETase variants*

*• NEQ free energy calculations accurately predict changes in T*_*m*_ *of DuraPETase*

*• The DuraPETase variants S223Y, S42M, and S61M have increased T*_*m*_

**Graphical Abstract:**

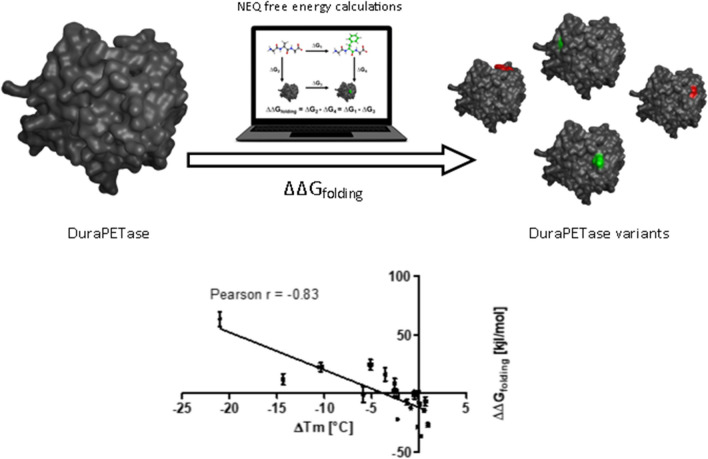

**Supplementary Information:**

The online version contains supplementary material available at 10.1007/s00253-024-13144-z.

## Introduction

Enhancing the thermostability of enzymes is a key aspect in the field of enzyme engineering as biotechnological and industrial application processes require highly stable enzymes (Bommarius et al. 2011; Nezhad et al. 2022). Directed evolution has proven to be a successful approach for developing thermostable proteins (Labrou 2010). It is, however, labor intensive and screening of a large number of enzyme variants is necessary. This makes this approach expensive and limits it to enzymes for which high-throughput assays are available. Computational methods that can aid in protein or enzyme design to reduce the screening efforts have therefore gained popularity. Software tools such as FoldX, RosettaDesign, or PoPMuSiC 2.1 make use of semi-empirical force fields to predict the change in folding free energy (∆∆*G*_folding_) of given mutations (Dehouck et al. 2009; Guerois et al. 2002; Kellogg et al. 2011). ∆∆*G*_folding_ gives the difference in ∆*G*_folding_ between a wild-type and a mutant protein, whereas ∆*G*_folding_ is the difference in free energy between the unfolded and the folded states. The prediction accuracy of these methods is however highly dependent on the investigated protein (Buß et al. 2018). Most of the time, a relatively high number of predictions still need to be tested to identify more stable variants (Wijma et al. 2014). Nevertheless, semi-empirical force field-based methods usually perform better than random selection of mutations (Buß et al. 2018). These predictions are often complemented by molecular dynamic (MD) simulations with subsequent analysis of the protein’s flexibility and/or hydrophobic surface area (Childers and Daggett 2017; Floor et al. 2014; Wang et al. 2018). The results attained from MD simulations are however difficult to analyze objectively, since analysis is dependent on correct visual inspection and interpretation of the MD trajectory (Wijma et al. 2014). In recent years, physically rigorous free energy calculations emerged as a promising new tool for the prediction of ∆∆*G*_folding_. When using this method to calculate ∆∆*G*_folding_ in a conventional, non-alchemical way, sampling of the complete folding or unfolding path of each variant is necessary. Even though this is possible in principle, it requires a lot of computational effort and is therefore often not feasible. Alchemical free energy calculations are physically rigorous calculations that circumvent this problem by estimating free energy differences along nonphysical, so-called alchemical paths that are more accessible to sampling by MD or Monte Carlo simulations. This way, alchemical free energy methods account for solvent effects, conformational changes, and electrostatic interactions with higher precision than semi-empirical methods. Several different approaches to alchemical free energy calculations have been developed and used in the past. Among the most popular is the free energy perturbation method based on the principles first published by Zwanzig (1954). Using this method, equilibrium simulations for several intermediate states along the alchemical path are simulated. From these, free energy differences are then estimated (Bennett 1976; Shirts and Chodera 2008). This approach has been implemented in the Schrödinger molecular modeling suite and has been used in several publications to accurately predict ∆∆*G*_folding_ or protein–protein interaction free energies upon amino acid mutation (Ford and Babaoglu 2017; Steinbrecher et al. 2017a, 2017b). It is, however, also possible to derive free energy differences from non-equilibrium simulations (NEQ) (Crooks 1999; Jarzynski 1997a, b). This was made easily accessibly within the GROMACS framework over the last couple of years by the group around de Groot (Gapsys et al. 2015b; Seeliger and de Groot 2010). This approach has been shown to give good prediction accuracy in retrospective studies, in which literature values were used to verify the validity of the predictions. Recently, the approach was also used prospectively to guide the functional analysis of a disease conferring mutation in the proliferating cell nuclear antigen followed by experimental validation (Hardebeck et al. 2023). Prospective, larger scale studies employing this method for the prediction of more thermostable enzyme variants, followed by experimental validation, are however scarce. We therefore decided to test the ability of a workflow based on NEQ alchemical free energy calculations to improve the thermal stability of a biotechnologically relevant enzyme. The development of solutions for the environmental burden of polyethylene terephthalate (PET) remains a daunting challenge (Yoshida et al. 2016). For the degradation of PET, the use of highly active biocatalysts is a promising approach. Since PET degradation is most efficient near the glass transition temperature of PET (ca. 70 °C), increasing the thermostability of the PET-degrading enzyme *Is*PETase is crucial for the efficient application of the enzyme in industrial processes (Jog 1995; Wei and Zimmermann 2017). Significant efforts have already been made to optimize and improve *Is*PETase (Bell et al. 2022; Brott et al. 2022; Liu et al. 2022; Lu et al. 2022; Shi et al. 2023; Zurier and Goddard 2023). DuraPETase is one of the most thermostable variants, with one of the highest PET degradation rates known today (Cui et al. 2021). It was created by experimentally testing and combining a library of 85 different mutations. This resulted in a thermostable enzyme variant of mesophilic PETase with ten additional point mutations and a *T*_m_ elevated by 31 to 78 °C. Further improvement of DuraPETase should therefore pose an interesting and challenging test case for the performance of the NEQ alchemical free energy approach.

Here, we present a study that utilizes NEQ alchemical free energy calculations prospectively to identify variants of DuraPETase with enhanced thermostability. We systematically evaluated the performance of two computational methods, Rosetta *ddg monomer* and NEQ free energy calculations, for the prediction of more thermostable variants of the highly thermostable DuraPETase. Calculations with Rosetta *ddg monomer* proved to be able to enrich stabilizing mutations. However, predictions did not significantly correlate with experimentally determined *T*_m_. ∆∆*G*_folding_ of 23 DuraPETase variants (Table S1) predicted by NEQ free energy calculations correlated well with experimentally determined *T*_m_ with a Pearson’s correlation coefficient of *r* = -0.84. Among these variants, two variants (DuraPETase^S61M^ and DuraPETase^S223Y^) had increased *T*_m_ by up to 1 °C compared to the original DuraPETase.

## Material and methods

### Plasmid construction, mutagenesis, expression, and protein purification

The sequence of DuraPETase (GenBank: GAP38373.1) was codon optimized for *E.* coli and synthesized by Twist Bioscience (South San Francisco, USA). The codon-optimized sequence of the DuraPETase gene is given in Sequence S1. It was inserted into the plasmid p15-mNeonGreen (Hardebeck et al. 2023) by restriction and ligation to create the plasmid pDuraPETase for intracellular expression of the original DuraPETase under control of a T7 promotor. Point mutations for the generation of DuraPETase variants were introduced by PCR via In-Fusion cloning (Takara Bio, Kusatsu, Japan). *E. coli* BL21(DE3) was used as the host for protein expression. Cells containing one of the respective plasmids for the expression of the original DuraPETase or its variants were grown in 500 ml lysogeny broth-Miller (LB) medium supplemented with 50 µg/ml carbenicillin at 33 °C and 160 rpm. Gene expression was induced with 1 mM IPTG as soon as an optical density at 578 nm of 0.8 was reached and incubation was continued for 24 h at 16 °C and 160 rpm. Cells were harvested by centrifugation (5000 × g, 10 min, 4 °C), suspended in 5 ml loading buffer (50 mM NaH_2_PO_4_, 300 mM NaCl, 10 mM imidazole, pH 8), and lysed using a sonicator. Cell debris were removed by centrifugation (48,000 × g, 15 min, 4 °C). The supernatant was used to isolate the respective enzyme via Ni–NTA affinity chromatography using Protino Ni–NTA agarose beads (Th. Geyer, Höxter, Germany), and the buffer was exchanged for storage buffer (50 mM Na_2_HPO_4_-Hcl, pH 7, 100 mM NaCl) by dialysis. Enzyme concentrations were determined by measuring the absorbance at 280 nm.

### Differential scanning fluorimetry (DSF)

To determine the apparent *T*_m_ of the original DuraPETase and its variants by DSF, 4 µM of the respective enzyme in 25 µl of PBS containing a total of 20 × SYPRO orange protein gel stain (Sigma-Aldrich, St. Louis, USA) and a Rotor-Gene Q 2 Plex HRM (Qiagen, Hilden, Germany) were used (Lavinder et al. 2009). The samples were heated from 30 to 95 °C, increasing the temperature by 0.5 °C every 5 s. SYPRO orange was excited at 470 ± 10 nm, and the emission was detected at 610 ± 5 nm. All measurements were performed three times from individually prepared samples. The apparent *T*_m_ were calculated from the extreme points of the first derivative of the fluorescence dF/DT(T) formed from the recorded melting curves as function F(T) using the Rotor-Gene Q software.

### Determination of PET degradation rates

The PET degradation rates for the original DuraPETase and its variants were tested using amorphous PET (2.4% crystallinity, determined by differential scanning calorimetry) with a thickness of 0.25 mm purchased from Goodfellow Cambridge Limited (Huntingdon, England). Substrate cut-outs with a diameter of 6 mm were incubated in 300 µl of 50 mM bicine NaOH buffer pH 9 containing 100 nM of the respective enzyme in microvolume reaction tubes. All reactions were performed as triplicates and were incubated at 50 °C or 52 °C and 300 rpm for 3 days. The reactions were stopped by removing the substrate. Three volumes of acetonitrile were added, and a centrifugation step (20,000 × g, 10 min) was performed. For each sample, 10 µl of the supernatant was injected into a LaChrom Elite HPLC system equipped with a L-2455 DAD detector (Hitachi, Chiyoda, Japan) and a Nucleodur C18 HTec column (Macherey–Nagel, Düren, Germany). The separation was performed isocratically at 40 °C with a flow rate of 0.5 ml/min for 8 min using 70% ddH_2_O, 20% acetonitrile, and 10% formic acid. The signal was detected at 254 nm, and standards of the reaction products terephthalic acid (TCI, Tokyo, Japan) and 2-hydroxyethyl terephthalic acid (Activate Scientific, Prien am Chiemsee, Germany) were used to quantify the degradation rate.

### Rosetta ddg monomer calculations

Rosetta version 3.12 was used for the *ddg monomer* calculations. The crystal structure of DuraPETase (PDB ID: 6ky5) was used for all calculations. Only chain A was retained. Ions and water molecules were deleted. First, minimization was performed using distance constrains on Cα atoms. The protocol described as “row 3” in Kellogg et al. was used for all calculations. This protocol was chosen, because it exhibited a high prediction accuracy for the dataset analyzed in the original publication (Kellog et al. 2011), while also appeared to be computationally inexpensive. The commands are described in detail in the Supporting information of Kellogg et al. (2011).

### NEQ alchemical free energy calculations

#### System preparation

The crystal structure of DuraPETase (PDB ID: 6ky5) was retrieved from the PDB server. Only chain A was retained. Ions and water molecules were deleted. Protonation states were assigned with the Protonate 3D function from MOE (Molecular Operating Environment, Chemical Computing Group, Canada version: 2022.02) using default settings at pH = 7.0. Mutations were generated using the Protein Builder. Sidechains were repacked and minimized using the Minimize tool, while a tether was applied to the backbone. The structures were exported from MOE in PDB format and used for MD system preparation within GROMACS 2019.3 (Abraham et al. 2015).

#### Free energy calculations and hybrid topology generation

For the estimation of the change in folding free energy (∆∆*G*_folding_) upon amino acid mutation, a well-established thermodynamic cycle was constructed according to Aldeghi et al. (2019). The cycle is also shown in Figure S1 for clarity. The alchemical transitions between both end states were performed along the ∆*G*_1_ arrow for the unfolded and along the ∆*G*_3_ arrow for the folded state. The unfolded state was modeled by a Gly-X-Gly tripeptide as done in previous publications (Gapsys et al. 2015b, 2016). All simulations were run independently from each other. The pmx package was used to generate hybrid topologies (Gapsys et al. 2015b). This was done after equilibrium simulations were performed. This way, equilibrium trajectories of the original DuraPETase could be used for different amino acid transitions.

#### Molecular dynamic (MD) simulations

GROMACS 2019.3 was used to carry out all MD simulations (Abraham et al. 2015). The Amber99SB*ILDN force field was used to model the protein (Lindorff-Larsen et al. 2010). Water molecules were represented by the TIP3P water model. Proteins or tripeptides were solvated in a cubic box with 150 mM of Na^+^ and Cl^−^ ions. The total charge of the system was neutralized with either Na^+^ or Cl^−^. For equilibrium simulations, energy minimization was performed using the steepest decent method. Equilibration was performed for 500 ps in the NVT and for 500 ps in the NPT ensemble. A position restraint with a value for the harmonic force constants of 1000 kJ mol^−1^ nm^−2^ was applied to all heavy atoms of the system during both equilibration phases. Production simulations were run for 10 ns. H-Bonds were constrained using LINCS algorithm (Hess et al. 1997), and the simulation temperature was controlled by the velocity rescaling thermostat at 300 K every 0.1 ps (Bussi et al. 2007). The Parrinello-Rahman barostat was used to control pressure at 1 atm (Parrinello and Rahman 1981). Particle mesh Ewald was used to treat electrostatic interactions in the simulation with a cutoff set to 1.2 nm (Essmann et al. 1995). The mean RMSD during the production stage of each replica was calculated to check the system stability.

After equilibrium simulation, snapshots were extracted to construct hybrid topologies and perform the NEQ transition simulations. To do so, the first 2.5 ns of each trajectory was discarded and 75 snapshots were extracted equidistantly from the remaining trajectory. The pmx package was used to generate hybrid topologies (Gapsys et al. 2015b). Transitions were performed in 100 ps after energy minimization and 50 ps of equilibration. The resulting change in *λ* was 5 × 10^−5^/step. A softcore potential with default parameters was applied to the Lennard–Jones and electrostatic interactions. Using the scripts provided with the pmx package, extracted work values were used to estimate the free energy at 300 K. The free energy estimation was based on the Crooks fluctuation theorem (Crooks 1999). Bennett acceptance ratio was used as a maximum likelihood estimator (Shirts et al. 2003). The uncertainty given is the standard deviation over multiple independent replicas. Either ten or four independent replicas were used at different stages of the study as stated in the “Results” section.

## Results

### In silico screening for stabilizing mutations in DuraPETase using ddg monomer

**Fig. 1:**
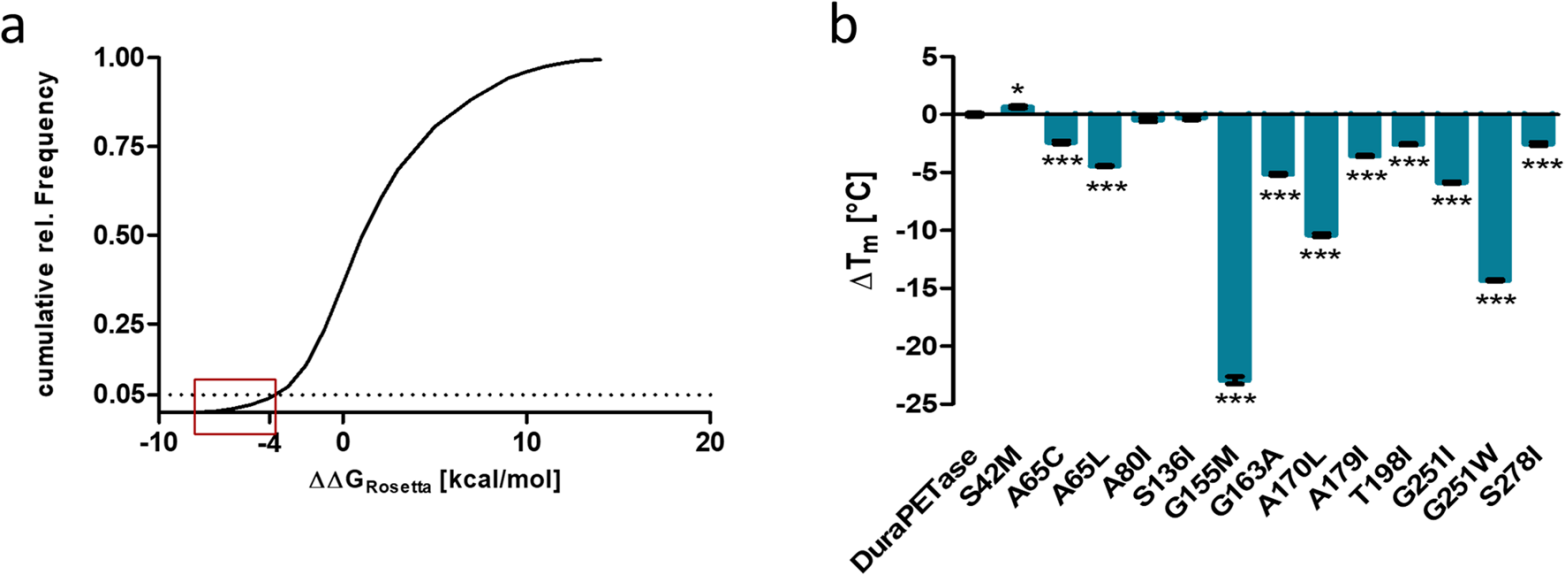
**a** Cumulative distribution of *ddg monomer* results predicting the change in folding free energy upon amino acid mutation of DuraPETase. Mutations from and to proline, mutations involving a net charge change, and mutations involving the catalytic triad were excluded. The red box indicates the 130 variants below the cutoff value of ∆∆*G*_Rosetta_ <  − 3.8 kcal/mol from which 13 were randomly selected for experimental validation. **b** ∆*T*_m_ were calculated by subtracting the *T*_m_ value of the original DuraPETase from the *T*_m_ value of each respective variant. *T*_m_ values were determined by DSF. The variants were randomly selected among the variants within the top 5% of predictions made with *ddg monomer*. Measurements were performed in triplicate. Statistical significance was analyzed by one-way ANOVA with Dunnett’s multiple comparison test. Single asterisk (*) corresponds to *p* < 0.05 and triple asterisk (***) corresponds to *p* < 0.001

The high computational demand of alchemical free energy calculations is one of its drawbacks and makes comprehensive screening of the entire mutational space of a protein practically impossible in the foreseeable future. Semi-empirical force field-based methods provide a fast alternative to systematically analyze single-point mutations of the entire protein. We therefore decided to include a first virtual screening step using the Rosetta *ddg monomer* tool in the optimization of thermostability of DuraPETase (Kellogg et al. 2011). This also provided the opportunity to test, whether further improvement of the thermostability of DuraPETase could be achieved using this simpler approach. A systematic screening of all possible single amino acid mutations, with some exceptions, was performed. Firstly, mutations from and to proline are not supported by the later employed NEQ free energy calculations at the moment. They were therefore excluded in this step. Secondly, mutations involving the catalytic triad of DuraPETase were also excluded, as these amino acids are essential for catalytic activity. Lastly, charge change mutations were shown to be poorly predicted by the ddg monomer approach (Gapsys et al. 2016). Charge change mutations also need to be setup in a slightly different way for the NEQ free energy calculations due to artifacts introduced by a net charge change. Additionally, even if properly set up, charge change mutations still require substantially longer to reach convergence during the simulation (Patel et al. 2021). Taking all of this into account, we decided to exclude charge change mutations from our screening. In the end, this resulted in a total of 2838 single amino acid mutations for the 264 amino acids comprising DuraPETase. The change in folding free energy of these mutations was calculated with *ddg monomer* employing the row 3 protocol from Kellogg et al. (2011). These calculations were completed in a single day on a medium-sized cluster. Mutations were then ranked according to their score, which is intended to correlate with ∆∆*G* but is not a quantitatively predicted change in folding free energy (Fig. 1a). The score calculated with *ddg monomer* will be named ∆∆*G*_Rosetta_ in the following to distinguish it from the ∆∆*G*_folding_ determined by NEQ alchemical free energy calculations. Cutoff values varying from <  − 0.75 to <  − 5 kcal/mol are often used to identify stabilizing mutations (Buß et al. 2018; Wijma et al. 2014). The cutoff for the mutational scan of DuraPETase was set to ∆∆*G*_Rosetta_ <  − 3.8 kcal/mol. This was within the normally chosen range of cutoff values and comprises the top 5% of the scores corresponding to 130 different DuraPETase variants. These variants should next be characterized experimentally. As our laboratory capacities did not allow for the purification and characterization of around 130 different DuraPETase variants, 13 variants were chosen randomly from below the threshold for experimental verification. Variants were purified by Ni–NTA affinity chromatography. The respective change in the apparent melting temperatures (Δ*T*_m_) for each variant compared to the original DuraPETase was calculated by substracting the *T*_m_ of the original DuraPETase from the *T*_m_ of the variant. Both *T*_m_ were measured by differential scanning fluorimetry (DSF) (Fig. 1b). Only a single variant, DuraPETase^S42M^, had a significantly higher *T*_m_ than the original DuraPETase. The *T*_m_ of the DuraPETase^S42M^ variant was 0.66 ± 0.17 °C higher. The *T*_m_ of DuraPETase^S136I^ and DuraPETase^A80I^ did not differ from the original DuraPETase. All other variants had significantly lower *T*_m_ when compared to the original DuraPETase. Consequently, the success rate was low. The prediction accuracy was also rather poor, as four of the mutations (DuraPETase^G155M^, DuraPETase^A170L^, DuraPETase^G251I^, and DuraPETase^G251W^) predicted to be stabilizing were destabilized by more than 5 °C. Due to the low prediction accuracy, a set of 13 DuraPETase variants with a wide range of *T*_m_ was obtained, providing a reasonable dataset that was used to test the accuracy of NEQ free energy calculations for the DuraPETase in a next step.

### Prediction accuracy of NEQ free energy calculations of DuraPETase

**Fig. 2:**
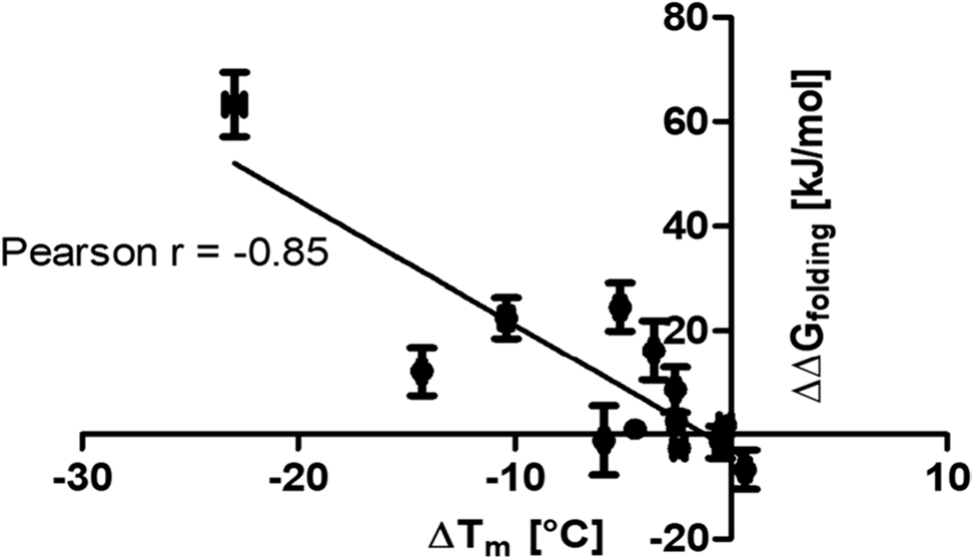
Experimentally determined ∆*T*_m_ plotted against ∆∆*G*_folding_ calculated by NEQ free energy calculations of 13 DuraPETase variants. ∆*T*_m_ were calculated by subtracting the *T*_m_ value of the original DuraPETase from the *T*_m_ value of each respective variant. *T*_m_ values were determined by DSF. The uncertainty given is the standard deviation of three independent measurements. The uncertainty of ∆∆*G*_folding_ for each mutation is the standard deviation of ten independent replicas. A significant negative correlation between ∆*T*_m_ and ∆∆*G*_folding_ was observed (Pearson *r* = -0.85, *p* = 0.002, *N* = 13). Linear regression (black line) gave an *R*^2^ = 0.70

In order to analyze how well the results from the experimental testing could be calculated with NEQ free energy calculations, these calculations were performed for all of the 13 variants that were tested experimentally in the first screening round (Fig. 1b). Ten independent calculations were performed for each amino acid mutation using equilibrium trajectories of 10 ns in order to obtain a reliable estimate of uncertainty. Previous research has shown that running several short simulations results in more accurate error estimates than running fewer long simulations (Bhati and Coveney 2022). Analysis of the mean RMSD of the original DuraPETase confirmed the stability of the system during equilibrium MD simulation (Supplementary Figure S2). The hybrid topologies for the NEQ transitions were constructed after running the equilibrium trajectories, as explained in the “Material and methods” section. This way, the equilibrium trajectories of the original DuraPETase could be used for all transitions, which substantially reduced computational cost. NEQ free energy calculations are easily scalable, as the many short non-equilibrium transitions can be run independently. This way, calculations could be performed relatively quickly in approx. 3 days, on a medium-sized cluster. Predicted ∆∆*G*_folding_ was obtained as the result of the calculation. Figure 2 shows the correlation between the predicted ∆∆*G*_folding_ and the experimentally determined ∆*T*_m_ of the 13 DuraPETase variants described above. A significant Pearson correlation between ∆∆*G*_folding_ and ∆*T*_m_ of *r* =  − 0.84 was determined. Linear regression gave an *R*^2^ = 0.70. A negative correlation was expected, as higher or more positive ∆∆*G*_folding_ values correspond to thermodynamically unstable proteins. These proteins have a lower *T*_m_ and therefore a negative ∆*T*_m_. Even though ten replicas were used for ∆∆*G*_folding_ calculations, the prediction accuracy for the 13 DuraPETase mutations also remained consistent when using only four randomly selected replicas. Therefore, it should be sufficient in this case to run only four replicas per prediction for further calculations to reduce the computational burden. It is worth noting that our dataset is biased towards predominantly destabilizing mutations. It is therefore especially encouraging that DuraPETase^S42M^, which is the only stabilizing mutation, is predicted with ∆∆*G*_folding_ =  − 6.7 kJ/mol, the lowest among the predictions. Overall, we concluded that NEQ free energy calculations can identify stabilizing mutations in DuraPETase.

### Two-step screening approach using ddg monomer and NEQ free energy calculations

**Fig. 3:**
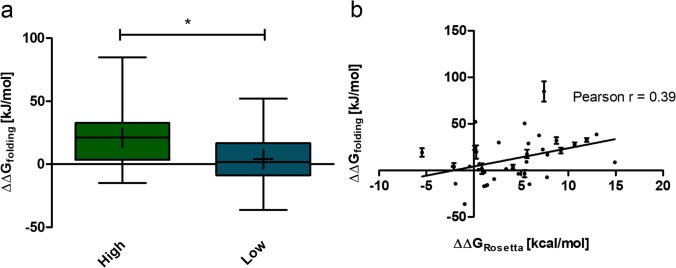
**a** ∆∆*G*_folding_ predictions calculated with NEQ free energy calculations of 19 single amino acid mutations from the *high* and 20 single amino acid mutations from the *low ddg monomer* prediction subsets. The *high* subset was defined as predictions with a ∆∆*G*_Rosetta_ > 4 kcal/mol. The *low* subset was defined as predictions with a ∆∆*G*_Rosetta_ < 4 kcal/mol. The mean ∆∆*G*_folding_ was significantly lower for the *low* subset (*p* = 0.013), as indicated by an asterisk. **b** ∆∆*G*_folding_ predicted by NEQ free energy calculations plotted against the ∆∆*G*_Rosetta_ for single amino acid mutations. The error given for ∆∆*G*_folding_ is the standard deviation over four independent replicas. A significant Pearson correlation (Pearson *r* = 0.39, *p* = 0.0145, *N* = 39) of the ∆∆*G*_Rosetta_ and ∆∆*G*_folding_ values was observed

Free energy changes calculated by NEQ free energy calculations showed a strong correlation with experimentally determined *T*_m_ but are computationally demanding. We therefore aimed to determine if the results of the *ddg monomer* calculations could serve as an initial screening tool to enrich promising mutations for further investigation. Twenty single amino acid mutations were selected from the *ddg monomer* prediction pool of 801 predictions having a ∆∆*G*_Rosetta_ exceeding 4 kcal/mol. Another 20 were selected from a pool of 2037 predictions with a ∆∆*G*_Rosetta_ lower than 4 kcal/mol. This cutoff was selected to test whether Rosetta *ddg monomer* could differentiate destabilizing mutations from stabilizing or neutral ones. The subsets were labelled “high” and “low,” respectively. NEQ free energy calculations were used to predict ∆∆*G*_folding_ for each individual amino acid mutation in both groups. The simulations for one mutation in the *high* subset did not converge properly, as evident by the insufficient overlap in the work distribution to estimate ∆G. This mutation was excluded from further analysis. Thus, the resulting dataset consisted of 39 data points. The mean ∆∆*G*_folding_ calculated with the NEQ alchemical free energy workflow was compared between the *high* and *low* datasets (Fig. 3a). The mean ∆∆*G*_folding_ for all predictions derived from the *high* subset was 21.18 ± 5.36 kJ/mol. In contrast, the mean ∆∆*G*_folding_ for all predictions from the *low* subset was 4.01 ± 4.53 kJ/mol. This difference was statistically significant (*p* = 0.020). The four most negative ∆∆*G*_folding_ predictions all belonged to the *low* subset. Subsequently, ∆∆*G*_folding_ values, which were calculated with the NEQ free energy protocol, were plotted against ∆∆*G*_Rosetta_ for every single amino acid mutation (Fig. 3b). The ∆∆*G*_Rosetta_ and ∆∆*G*_folding_ were significantly correlated with Pearson’s correlation coefficient of *r* = 0.39. Consequently, ∆∆*G*_Rosetta_ can provide a rough estimation of the impact of a single amino acid mutation on protein stability. This suggests that *ddg monomer* can serve as an initial enrichment tool for identifying potentially favorable mutations. This way, the computational effort for the identification of potentially stabilizing mutations with NEQ free energy calculations may be reduced.

### Identification of stabilizing mutations using ddg monomer and NEQ free energy calculations

**Fig. 4:**
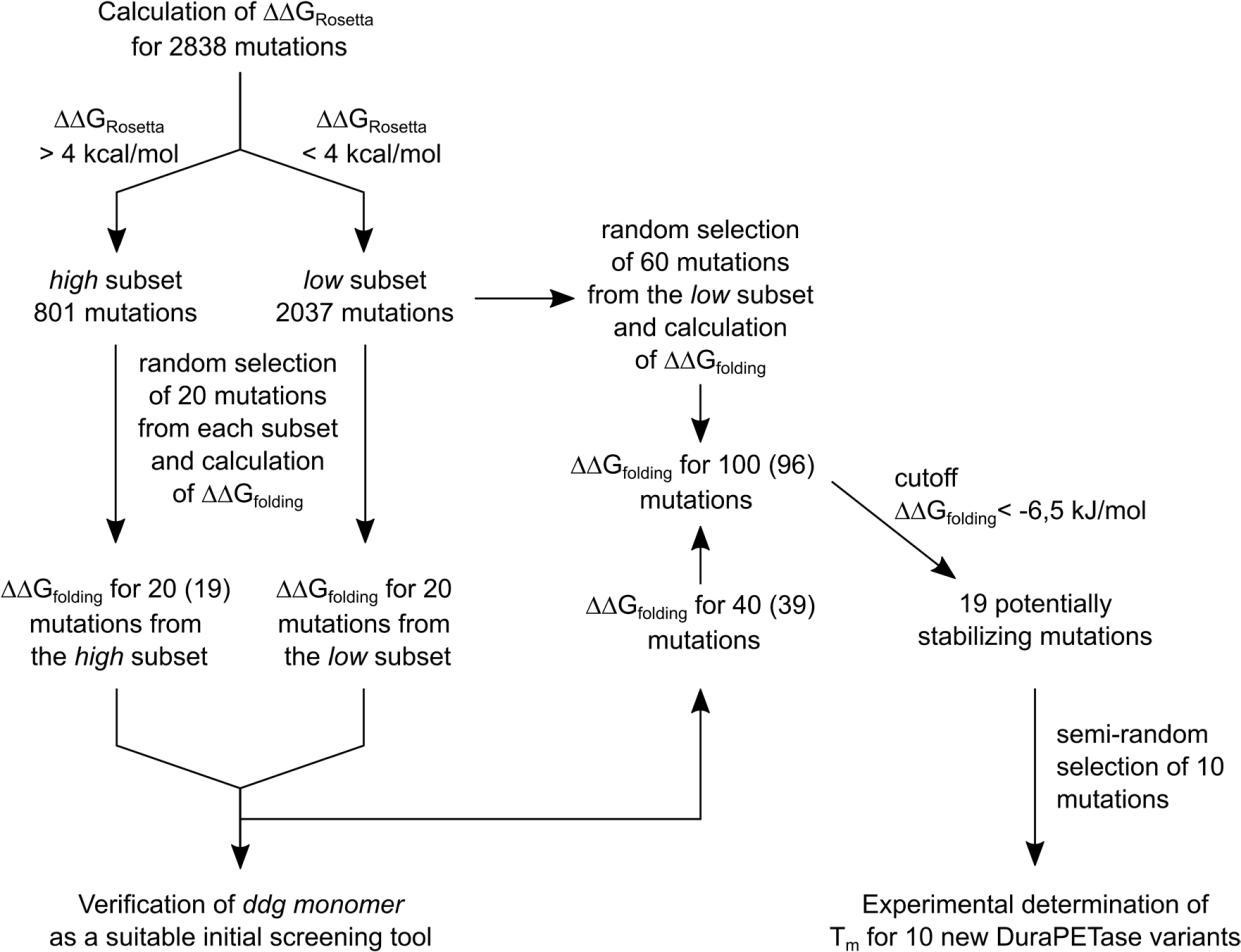
Flow diagram of the complete workflow used in this study for the identification of more thermostable DuraPETase variants. Numbers shown in brackets show the actual number of successful run predictions, if it deviated from the initially intended number due to incomplete convergence in some of the predictions

**Fig. 5:**
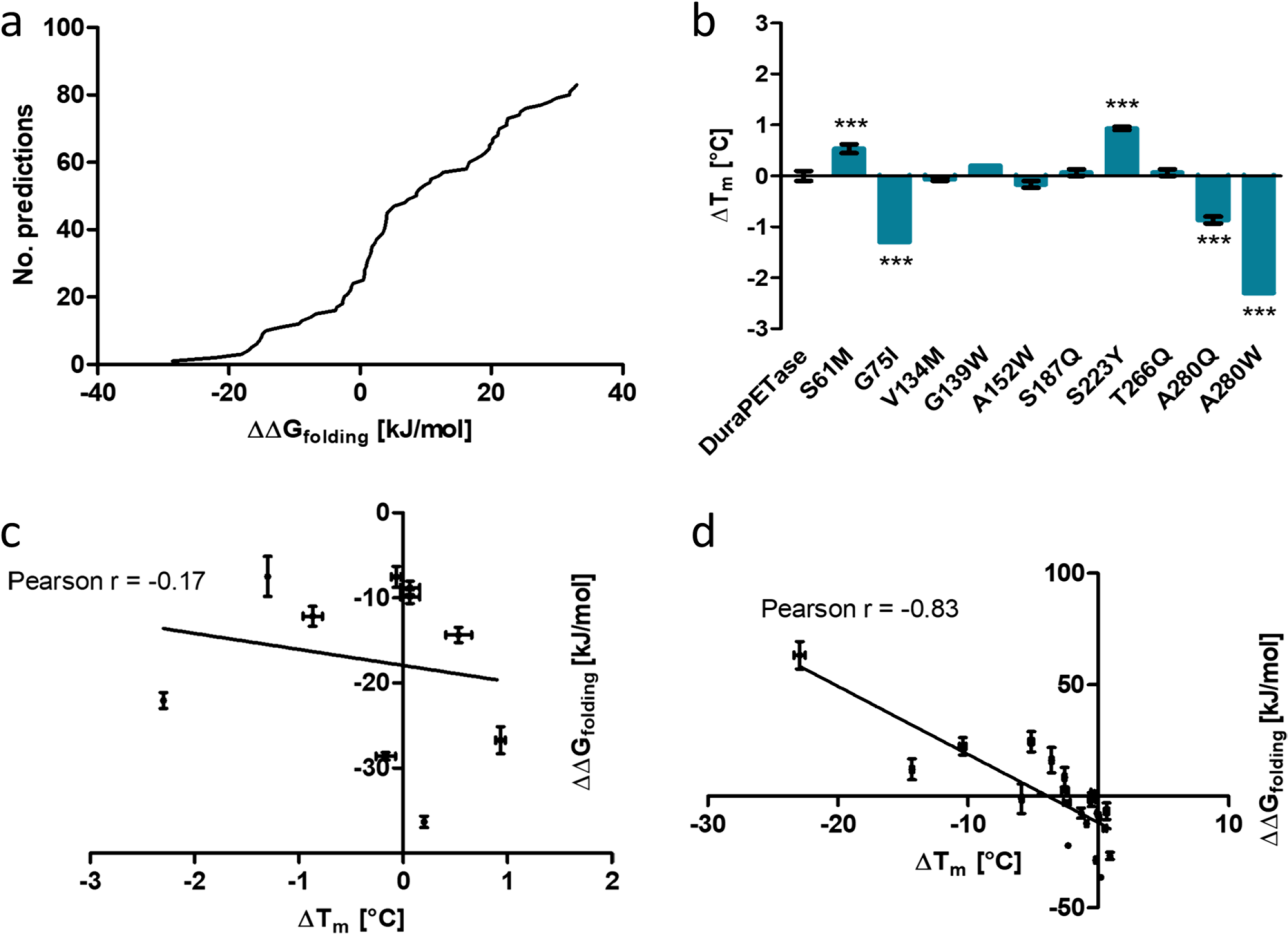
**a** Cumulative distribution of ∆∆*G*_folding_ calculated with NEQ free energy calculations for single amino acid mutations of DuraPETase. Eighty mutations were selected from the *low* subset of predictions from the *ddg monomer* results, while 20 were chosen from the *high* subset. For four mutations, insufficient overlap in the work distribution for estimating ∆*G* was observed. They were excluded from further analysis. In total, the results of 96 predictions are shown. **b** Δ*T*_m_ of different purified DuraPETase variants. ∆*T*_m_ were calculated by subtracting the *T*_m_ value of the original DuraPETase from the *T*_m_ value of each respective variant. *T*_m_ values were determined by DSF. The variants were selected among mutations predicted to have ∆∆*G*_folding_ less than − 6.5 kJ/mol calculated with NEQ free energy calculations. **c** Experimentally determined ∆*T*_m_ values plotted against ∆∆*G*_folding_ values calculated by NEQ free energy calculations of ten DuraPETase variants from the second round of screening. No significant correlation of the ∆*T*_m_ values and ∆∆*G*_folding_ values was observed (Pearson *r* = -0.17, *p* = 0.63, *N* = 10). **d** Experimentally determined ∆*T*_m_ values plotted against ∆∆*G*_folding_ values calculated by NEQ free energy calculations of 23 DuraPETase variants from both rounds of screening. A significant negative correlation of ∆*T*_m_ values and ∆∆*G*_folding_ values was observed (Pearson *r* = -0.83, *p* < 0.0001, *N* = 23). The uncertainty given for ∆*T*_m_ is the standard deviation of three independent measurements. Statistical significance was analyzed by one-way ANOVA with Dunnett’s multiple comparison test. Triple asterisk (***) corresponds to *p* < 0.001. The uncertainty of ∆∆*G*_folding_ for each mutation is the standard deviation of four independent replicas

In the first two parts of this study, a model was built that could accurately predict the change in thermostability of DuraPETase upon single amino acid mutation. In the third part, it was also shown that *ddg monomer* calculations can be used to enrich promising mutations. We now decided to utilize these findings prospectively, to identify stabilizing mutations in addition to DuraPETase^S42M^. To do so, ∆∆*G*_folding_ of another 60 variants was predicted. The entire workflow including the verification steps is summarized in Fig. 4. In accordance with the results presented above, the variants were randomly selected from the *low* group of *ddg monomer* predictions. Together with 40 already calculated mutations (Fig. 3), a total of 100 predictions were run. Out of these 100, the results of the calculations for four mutations could not be analyzed properly because the overlap in the work distribution for estimating ∆*G* was insufficient. In the end, this resulted in 96 successfully calculated ∆∆*G*_folding_ predictions (Fig. 5a). The top ranking 11 mutations all were mutations to aromatic amino acids. To minimize potential bias, we decided to choose variants for experimental validation from among the top predictions as follows. A cutoff value of ∆∆*G*_folding_ =  − 6.5 kJ/mol that corresponds to the calculated ∆∆*G*_folding_ of the already identified stabilizing DuraPETase^S42M^ variant was chosen. Nineteen variants were below this cutoff value. Ten variants were selected semi-randomly from these variants. However, the number of mutations to each single amino acid was limited to a maximum of three. The selected variants were expressed and purified and the *T*_m_ was measured by DSF (Fig. 5b). Two variants had significantly higher *T*_m_ than the original DuraPETase. Five variants had the same *T*_m_ as the original DuraPETase, while three variants had lower *T*_m_. DuraPETase^S223Y^ was the most stable among all tested variants. The *T*_m_ was 0.93 °C higher than that of the original DuraPETase. DuraPETase^A280W^ had a *T*_m_ that was 2.30 °C lower than that of the original DuraPETase. It was the most unstable variant. The entire range of determined *T*_m_ from this screening was only 3.43 °C. This is distinctly smaller than in the first screening round that was based on *ddg monomer* predictions, which had a range of *T*_m_ of 21.66 °C. The number of mutations that were identified to be stabilizing with the second screening round was higher, two compared to one, even though the number of enzymes tested was smaller (10 instead of 13). There was however no significant correlation between the ∆*T*_m_ values and ∆∆*G*_folding_ for the variants from the second round screening (*r* =  − 0.17, *p* = 0.63, *N* = 10) (Fig. 5c). ∆*T*_m_ values of all 23 experimentally tested mutations were plotted against the calculated ∆∆*G*_folding_ (Fig. 5d). This resulted in a Pearson correlation of *r* =  − 0.82, which is slightly worse than the correlation determined for the first dataset alone. This shows that the prediction accuracy over the entire range of mutations is very good. When only focusing on stabilizing mutations, the prediction accuracy drops however. The lower accuracy of the predictions for stabilizing mutations also explains the slightly lower correlation of the entire dataset, when compared to the mutations from the first screening round alone. These results indicate that NEQ calculations predict ∆∆*G*_folding_ accurately as long as the mutation is not strongly stabilizing. With mutations predicted to be stabilizing, the effect seems to be overestimated. Stabilizing or neutral mutations are nevertheless enriched by the NEQ free energy calculations.

### PET hydrolysis by DuraPETase variants

**Fig. 6:**
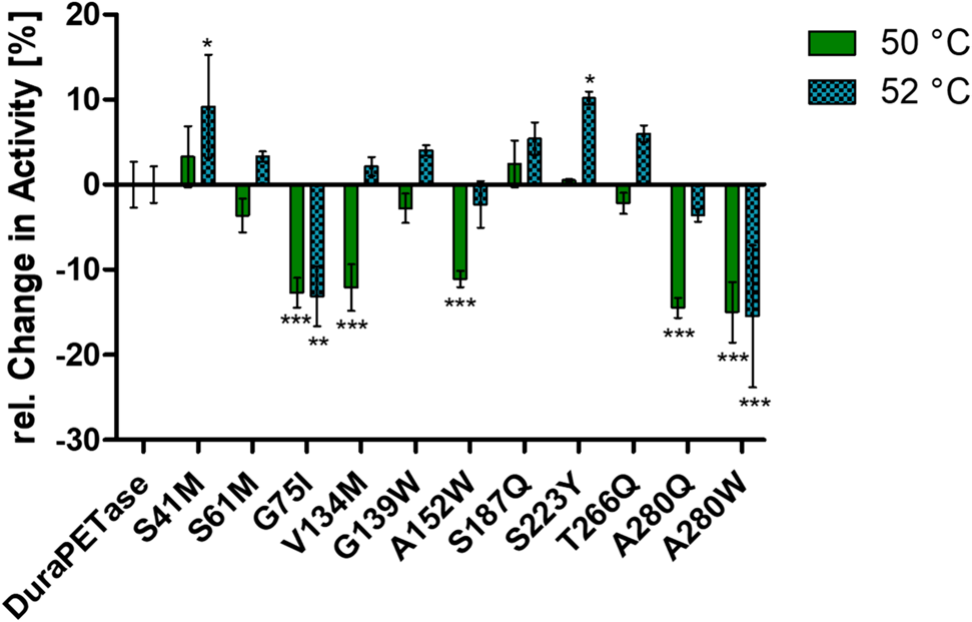
Relative activity alterations of DuraPETase variants at 50 °C and 52 °C in comparison to the original DuraPETase. The purified enzymes were used at 100 nmol/l and incubated for 3 days at 50 °C or 52 °C in 300 µl of 50 mmol/l bicine NaOH buffer pH 9 with a 6 mm diameter cutout of amorphous PET. The PET degradation activity was quantified by determining the concentration of the reaction products by HPLC. The changes in activity are given as relative values and are normalized to the PET degradation rates for the original DuraPETase at 50 °C and 52 °C, respectively. All experiments were performed as triplicates. Statistical significance was analyzed by one-way ANOVA with Dunnett’s multiple comparison test against the DuraPETase sample at the corresponding temperature. Triple asterisk (***) corresponds to *p* < 0.001, double asterisk (**) corresponds to *p* < 0.01, and single asterisk (*) corresponds to *p* < 0.05

In addition to the already determined *T*_m_ for the ten DuraPETase variants from the second round of screening, their respective activities on amorphous PET were analyzed as well (Fig. 6). Since the variant DuraPETase^S42M^ from the first round was found to be stabilizing, it was also included. The activity was tested at 50 °C and 52 °C to see how the enzymes performed at the optimum temperature of the original DuraPETase (50 °C) and slightly above this temperature. Overall, the changes in activity at 50 °C and 52 °C reflect the observed changes in thermostability. DuraPETase^S223Y^—which showed the strongest increase in T_m_—showed practically identical PET degradation rates at 50 °C compared to the original DuraPETase but was approx. ten percent more active at 52 °C. Similarly, a better performance at 52 °C was also recorded for one of the other two more thermostable variants, DuraPETase^S42M^. For the third stable variant, DuraPETase^S61M^, the same tendency was observed at 52 °C, the results were however not statistically significant. The variants DuraPETase^G75I^, DuraPETase^A280W^, and DuraPETase^A280Q^ all showed a reduced thermostability. In line with this, there was a pronounced decrease of PET degradation rates at 50 °C for these variants. The overall changes in thermostability of the new variants were relatively small compared to the already very high *T*_m_ of original DuraPETase. In line with this, only relatively small changes could be measured when analyzing the PET degradation activity at a temperature slightly above the original optimum temperature.

## Discussion

In this study, a workflow for the improvement of enzyme thermostability based around the Rosetta *ddg monomer* tool and NEQ alchemical free energy calculations was developed. DuraPETase, the enzyme optimized in this study, is a highly evolved thermostable enzyme (Cui et al. 2021) and therefore an interesting test case for the performance and limitations of the NEQ alchemical free energy calculation approach. Alchemical free energy calculations have been established as a powerful tool for predicting the change in protein stability upon amino acid mutation (Gapsys et al. 2016; Steinbrecher et al. 2017b). They therefore provide the opportunity to reduce the number of enzyme variants that need to be tested experimentally. The high computational demand of free energy calculations has however limited their use in prospective studies.

This study demonstrates how to integrate free energy calculations into a protein engineering project to supplement well-established semi-empirical in silico methods (Bell et al. 2022; Cui et al. 2021). The use of the *ddg monomer* tool in the first round of screening resulted in the identification of a single DuraPETase variant with higher thermostability among 13 tested variants. Compared with previous results also using semi-empirical methods, this is on the lower end of successful predictions (Buß et al. 2018; Wijma et al. 2018). The outcome was however potentially better than the expected 2% success rate in a directed evolutionary approach (Bromberg and Rost 2009). Due to the rather small sample size, this is just a rough approximation and cannot be judged reliably. The NEQ alchemical free energy calculations performed next, resulted in a very good correlation between the experimentally measured *T*_m_ and the predicted ∆∆*G*_folding_. Pearson’s correlation coefficient was − 0.85. Linear regression gave an *R*^2^ = 0.73. Notably, *T*_m_ is not a direct measure for ∆*G*_unfolding_ at standard conditions (Peccati et al. 2023). Nevertheless, the correlation between *T*_m_ and ∆*G*_unfolding_ generally is extremely high (Gapsys et al. 2016; Wijma et al. 2014). Pearson’s correlation coefficient for the Δ*T*_m_ and changes in folding free energy falls within the same range as the previously reported excellent correlation of *r* = 0.86 determined with the same approach (Gapsys et al. 2016). Studies employing equilibrium free energy perturbations (FEP) reported on slightly worse *R*^2^ of 0.4 and 0.65 compared to the *R*^2^ of 0.73 reported in this study for the correlation between experimentally and predicted ∆∆*G*_unfolding_ (Jespers et al. 2019; Scarabelli et al. 2022; Steinbrecher et al. 2017b). One reason for the high correlation between *T*_m_ and ∆∆*G*_unfolding_ in our dataset might be the fact that we excluded charge changing mutations from our predictions. Charge change mutations have been shown to converge slower and are predicted less accurately by both NEQ and EQ alchemical free energy calculations (Clark et al. 2019; Patel et al. 2021). Purposefully omitting them might therefore increase the prediction accuracy.

With the intent to combine the *ddg monomer* tool and NEQ alchemical free energy calculations in the second screening round, it was first shown that the *ddg monomer* tool can enrich stabilizing mutations. Selection of mutations from a subset of possible mutations with lower ∆∆*G*_Rosetta_ led to an on average lower ∆∆*G*_folding_ predicted by NEQ alchemical free energy calculations. This shows that mutations with lower scores from the *ddg monomer* tool should be prioritized for free energy calculations. This two-step approach has the potential to save significant amounts of computational time while simultaneously enabling the screening of large portions of the sequence space. Out of a total of 96 NEQ alchemical free energy calculations, 19 were below a cutoff of − 6.5 kJ/mol. Out of these 19, 11 were mutations to aromatic amino acids. These mutations are generally considered to be stabilizing (Serrano et al. 1991). Aromatic amino acids thermodynamically favor the folded state of a protein, as this minimizes the solvent exposed hydrophobic surface area and enables hydrophobic interactions between the amino acids. It is however also possible that the stabilizing effect of aromatic amino acids might be overestimated by the NEQ alchemical free energy calculations, due to insufficient sampling or force field inaccuracies. This is also why we limited the experimental validation of variants to a maximum of three variants for a mutation into any single amino acid.

Ten of the variants predicted to be stabilizing in the second screening round were tested experimentally. No significant correlation between experimental and predicted values was observed for these ten variants. The prediction accuracy over the entire dataset of 23 mutations was however still good, with a Pearson’s correlations coefficient of *r* =  − 0.83. This indicates a better performance of the NEQ alchemical free energy calculations for destabilizing and neutral mutations and a worse prediction accuracy for stabilizing ones. In contrast to these findings, previous studies have shown that the correlation between experimental and predicted ∆∆G_folding_ is roughly the same for stabilizing and destabilizing mutations (Gapsys et al. 2016; Steinbrecher et al. 2017b). Naturally occurring proteins, as used in the datasets of theses previous studies, are most often mesophilic and only marginally stable (Goldenzweig and Fleishman 2018; Taverna and Goldstein 2002). DuraPETase, with its *T*_m_ of 78 °C, is however already highly thermostable. From this, the question arises, if the performance of NEQ free alchemical free energy calculations is worse for single amino acid mutations that further increase the stability of already highly stable proteins. Theoretically, this should not be the case, as NEQ alchemical free energy calculations present a physically rigorous way of calculating changes in protein folding free energy. Insufficient sampling during the MD simulation can however influence the accuracy of the predictions. Highly stable proteins are often more rigid, and transitions between relevant conformations might take more time, because they are separated from each other by higher energy barriers. Thorough sampling of all relevant conformations for highly stable proteins might therefore take more time than for less stable ones. This directly impacts the accuracy of the free energy prediction, as it is dependent on a correct frequency distribution of all relevant conformations. Performing multiple independent replicas, as done in this study, should provide a first indication if insufficient sampling poses a problem in the system under investigation. If, however, the number of replicas is too small and relevant conformations are only marginally populated, this approach might nevertheless provide a false error estimate. A convenient way to detect such limitations during MD sampling could be the usage of closed thermodynamic cycles with relative free energy differences at their edges. If the sum of all relative free energy changes deviates from zero, insufficient sampling for one or multiple of the mutations could be an issue (Gapsys et al. 2015a; Hardebeck et al. 2023). Both approaches mentioned above, the use of closed thermodynamic cycle and increasing the number of independent replicas, come however with a substantial increase in computational effort and need to be considered carefully. Aside from potential sampling issues, force field parametrization also influences the performance of NEQ alchemical free energy calculations (Gapsys et al. 2016). The force fields used in molecular simulations are typically parameterized based on experimental data and may not fully capture the nuances of highly stable proteins (Lindorff-Larsen et al. 2010). To the best of our knowledge, a comprehensive investigation on the performance of force fields on highly stable proteins has not been undertaken thus far. This could be an interesting topic for future research in the context of the application of free energy calculations.

Overall, we managed to identify three mutations that increased the thermostability of DuraPETase. The increase in thermostability was however limited, especially when compared with other optimizations of IsPETase (Bell et al. 2022; Cui et al. 2021; Lu et al. 2022). It is also a rather limited number of successfully identified mutations when compared to another protein engineering project. Song et al. used the well-established FoldX tool in conjunction with a free energy perturbation approach for the engineering of a more thermostable blue light photo receptor YtvA LOV domain from *Bacillus subtilis*. They identified nine stabilizing mutations and had a good correlation of *r* = 0.63 between experimentally determined and predicted ∆∆*G* values (Song et al. 2013). When one however considers the free energy surface as a function of protein sequence space, it is conceivable that several local minima exist in addition to the global minimum. If the sequence of DuraPETase represents a point close to such a local minimum on the energy hypersurface, only small increases in thermostability can be achieved by single amino acid mutations. This is consistent with reports in the literature where larger enhancement in the thermostability of DuraPETase was only achieved with the introduction of a disulfide bond or a salt bridge (Brott et al. 2022; Lu et al. 2022). Both are mutations that could not have been identified with our screening approach.

The three mutations identified with the screening approach with a higher *T*_m_ than original DuraPETase were DuraPETase^S42M^, DuraPETase^S61M^, and DuraPETase^S223Y^. None of these mutations are located near the active center. Interestingly, in the thermostable PETase variant HotPETase, the serine in position 61 was also mutated, however in this case to a valine (Bell et al. 2022). Both mutations introduce more lipophilic amino acids. The serine is located near the surface of the enzyme in a relatively hydrophilic region. Introduction of a hydrophobic residue in this region likely leads to a more compact packing of the region to reduce the surface exposed hydrophobic area. This could pose a possible explanation for the increased thermostability of the enzyme. The same explanation can be given for DuraPETase^S61M^ and DuraPETase^S223Y^. Serine 61 and serine 223 are also located near the surface of the enzyme, and mutation to a methionine or tyrosine also drastically increases the lipophilicity at these positions. Analysis of PET hydrolysis by the DuraPETase variants showed that variants with an improved thermostability also were more active than the original DuraPETase at an elevated reaction temperature of 52 °C. The most stable variant DuraPETase^S223Y^ showed the highest amount of PET degradation at 52 °C with a 10% increase compared to the original DuraPETase. If this can be attributed to an increased activity or a longer half-life of the enzyme at 52 °C remains unclear. Unfortunately, this slight increase in both the *T*_m_ and the improved degradation rate at 52 °C of the variant is likely not relevant for the potential industrial application of DuraPETase.

In conclusion, we demonstrated the applicability of NEQ alchemical free energy calculations for enzyme engineering projects. Challenges encountered with the extremely stable DuraPETase in this study, however, also highlight limitations of the approach and provide directions for the further optimization and validation of free energy calculations. We also validated an efficient way to integrate NEQ alchemical free energy calculations with semi-empirical methods to reduce overall computational cost. Overall, this study shows that free energy calculations can be a valuable tool for the design of thermostable proteins that can be readily employed in these kinds of projects.

## Supplementary Information

Below is the link to the electronic supplementary material.

Supplementary file1 (PDF 233 KB)

## Data Availability

Data is available from the corresponding author upon reasonable request.
