# Supplementary material for: Application of an alchemical free energy method for the prediction of thermostable DuraPETase variants
Source: Appl Microbiol Biotechnol. 2024 Apr 21;108(1):305. doi: 10.1007/s00253-024-13144-z (PMC11033240; doi:10.1007/s00253-024-13144-z)
Supplement: Supplementary file 1 — Supplementary file1 (PDF 233 KB) [file 253_2024_13144_MOESM1_ESM.pdf]

Applied Microbiology and Biotechnology

Application of an alchemical free energy method for the prediction of thermostable DuraPETase variants

Sebastian Schreiber[#], David Gercke[#], Florian Lenz, Joachim Jose*

University of Münster, Institute of Pharmaceutical and Medicinal Chemistry, PharmaCampus, Corrensstr. 48, 48149 Münster, Germany

[#] These authors contributed equally

*Corresponding author: joachim.jose@uni-muenster.de

Phone: +49 (0)251 8332200

Fax: +49 (0)251 8332210

Sequence S1: Gene sequence of DuraPETase with terminal XhoI/KpnI restriction sites codon optimized for *E. coli*:

```
ATGCTCGAGCAGACCAATCCGTATGCACGTGGTCCGAATCCGACCGCAGCAAGCCTGGAAGCAAGCGCAGGTCCG
TTTACCGTTCGTAGCTTTACCGTTAGCCGTCCGAGCGGTTATGGTGCAGGCACCGTTTATTATCCGACCAATGCC
GGTGGCACCGTTGGTGCAATTGCCATTGTTCCGGGTTATACCGCACGTCAGAGCAGCATTAAATGGTGGGGTCCG
CGTCTGGCAAGCCATGGTTTTGTTGTTATTACCATTGATACCAACAGCACCTTCGATTATCCGAGCAGCCGTAGC
AGCCAGCAGATGGCAGCACTGCGTCAGGTTGCCAGCCTGAATGGTGATAGCAGCAGCCCGATTTATGGTAAAGTT
GATACAGCACGTATGGGTGTTATGGGTCATAGCATGGGTGGTGGTGCAAGCCTGCGTAGCGCAGCAAATAATCCG
AGCCTGAAAGCAGCAATTCCGCAGGCTCCGTGGGATAGCCAGACCAATTTTAGCAGCGTTACCGTTCCGACACTG
ATTTTTGCATGTGAAAATGATAGCATTGCACCGGTTAATAGCCATGCACTGCCGATCTATGATAGTATGAGCCGT
AATGCAAAACAGTTTCTGGAAATTAATGGTGGTAGCCATAGCTGTGCAAATAGCGGTAATAGCAATCAGGCACTG
ATCGGTAAAAAAGGTGTTGCATGGATGAAACGCTTCATGGATAATGATACCCGTTATAGCACCTTTGCCTGCGAA
AATCCGAATAGCACCGCAGTTAGCGATTTTCGTACCGCAAATTGTAGCGGTACC
```

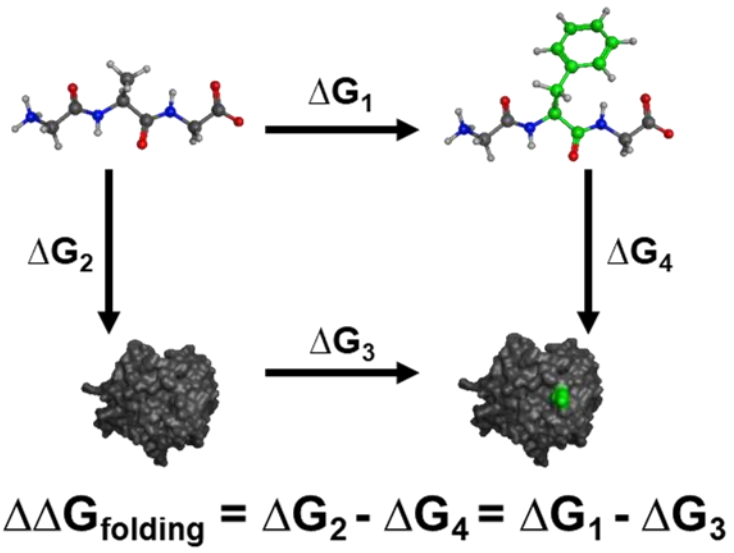

Fig. S1 Thermodynamic cycle for the estimation of the change in folding free energy ($\Delta\Delta G_{folding}$). Folding of the protein normally occurs along the ΔG_2 and ΔG_4 arrows. The alchemical transitions between both end states were performed along the ΔG_1 arrow for the unfolded and along the ΔG_3 arrow for the folded state.

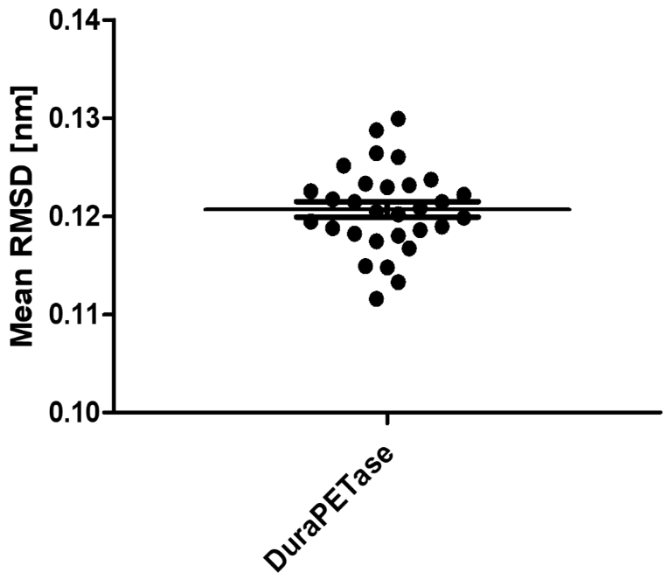

Fig. S2 Mean RMSD of 10 ns MD simulation production runs of 30 independent replicas of the original DuraPETase. To check that the system remained stable during 10 ns of MD simulation the mean RMSD of each of 30 different replicas was calculated.

Table S1 Computed and determined thermodynamic parameters of all experimentally tested DuraPETase variants

Variant	$\Delta\Delta G_{rosetta}$ [kcal/mol]	$\Delta\Delta G_{folding}$ [kJ/mol]	T_m [°C]
**DuraPETase**	n.d.	n.d.	78.3
**S42M**	-4.6	-6.7	79.1
**S61M**	-1.9	-14.4	78.8
**A65C**	-4.6	-2.6	76.0
**A65L**	-4.6	1.1	74.0
**G75I**	7.7	-7.5	77.0
**A80I**	-4.9	-1.5	78.0
**V134M**	0.0	-7.5	78.2
**S136I**	-4.4	1.8	78.2
**G139W**	-1.0	-36.3	78.5
**A152W**	2.0	-28.6	78.1
**G155M**	-4.6	63.3	68.0
**G163A**	-4.6	24.5	73.3
**A170L**	-5.8	22.4	68.0
**A179I**	-5.7	16.2	74.9
**S187Q**	-0.3	-9.8	78.4
**T198I**	-5.1	8.6	75.9
**S223Y**	0.0	-26.7	79.2
**G251I**	-5.4	-1.1	72.6
**G251W**	-6.4	12.1	64.1
**T266Q**	2.2	-8.8	78.4
**S278I**	-4.9	2.5	75.9
**A280Q**	0.8	-12.1	77.4
**A280W**	-0.1	-22.0	76.0
